# Attributed network embedding based on self-attention mechanism for recommendation method

**DOI:** 10.1038/s41598-023-44696-1

**Published:** 2023-11-01

**Authors:** Shuo Wang, Jing Yang, Fanshu Shang

**Affiliations:** Harbin Engineering University, No. 145 Nangang District, Harbin City, 150000 Heilongjiang Province China

**Keywords:** Computer science, Information technology

## Abstract

Network embedding is a technique used to learn a low-dimensional vector representation for each node in a network. This method has been proven effective in network mining tasks, especially in the area of recommendation systems. The real-world scenarios often contain rich attribute information that can be leveraged to enhance the performance of representation learning methods. Therefore, this article proposes an attribute network embedding recommendation method based on self-attention mechanism (AESR) that caters to the recommendation needs of users with little or no explicit feedback data. The proposed AESR method first models the attribute combination representation of items and then uses a self-attention mechanism to compactly embed the combination representation. By representing users as different anchor vectors, the method can efficiently learn their preferences and reconstruct them with few learning samples. This achieves accurate and fast recommendations and avoids data sparsity problems. Experimental results show that AESR can provide personalized recommendations even for users with little explicit feedback information. Moreover, the attribute extraction of documents can effectively improve recommendation accuracy on different datasets. Overall, the proposed AESR method provides a promising approach to recommendation systems that can leverage attribute information for better performance.

## Introduction

With the arrival of the Web 2.0 era, the problems of data explosion and data scarcity coexist, which pose a challenge for users to find valuable information in massive data. This problem has led to obstacles for users to obtain specific information and low data utilization, becoming one of the important issues in the development of big data. To address this challenge, recommendation systems have been widely applied and become one of the key methods to solve this problem. Recommendation systems^1^ can filter valuable information from massive data and provide feedback to users, thus improving data utilization while meeting personalized needs. Recommendation systems have become one of the most widely used methods in the field of information filtering, playing an important role in major internet platforms such as Amazon and Twitter. The widespread application of recommendation systems has proven their importance in meeting user needs and increasing user stickiness. At the same time, recommendation systems have also brought huge commercial profits to businesses. Therefore, recommendation systems have been widely studied and applied due to their good commercial value.

The core of the recommendation system is the recommendation algorithm, which generally conducts biased learning through the user's historical data, and learning needs to have enough historical interactive information. Collaborative filtering algorithm (CF) is to predict the recommendation results through the rating matrix of users and items, which first appeared in the GroupLens news recommendation system^2^. However, the rating matrix is often sparse, and the recommendation effect is often poor only through the rating matrix. At present, the Internet platform generally adopts the hybrid recommendation method, which combines the collaborative filtering algorithm with the content-based algorithm (CB) and fuses the auxiliary information to improve the performance of the collaborative filtering algorithm. For example, Park et al. use the background information of new users as auxiliary information to improve the cold start recommendation accuracy in the adaptive learning environment^3^. The SoRec model proposed by Ma et al.^4^ in 2008 introduced users' social relationships into recommendation. Hafed et al.^5^ proposed a new scenario-based collaborative filtering recommendation algorithm, introduced the concept of item scenario similarity to improve the similarity calculation equal, and finally used the clustering matrix and user's rating matrix to generate recommendation list. With the advancement of deep learning^6–12^, recent research has primarily focused on improving the framework of Graph Neural Networks (GNNs) and integrating multi-modal information. For example, the MKGAT model^13^, based on the KGAT model^14^, introduces multi-modal nodes by incorporating multi-modal features as nodes representing items into the knowledge graph. It utilizes Graph Attention Networks (GAT)^15^ to aggregate high-order neighbor information and obtain superior feature representations. However, constructing a comprehensive multi-modal knowledge graph may introduce noise and impose limitations on improving recommendation performance. Additionally, if a multi-modal knowledge graph is constructed for text-image training, it requires adding text and image node information for each item node, increasing the complexity of information requirements.

To address these challenges, the MGAT model^16^ first utilizes user-item interaction records and pre-trained modality-specific features to construct a unimodal graph for each modality. Subsequently, it employs Graph Attention Networks (GAT) and Gated Recurrent Units (GRU) to learn high-order neighbor information within the graph and capture local semantic information, further enhancing recommendation performance. However, this model primarily focuses on mining fine-grained user preferences and does not effectively address the issue of data sparsity.

Now the recommendation algorithm is gradually applied to some emerging business models, such as "virtual try on", "service robot", and so on. It is not difficult to find that the auxiliary information of users, such as personal data, that can be obtained and analyzed from such scenarios is very limited, which not only makes it very difficult to model users, but also often accompanied by the problem of cold start. Take the service robot as an example, the service person faces the public, so it is often necessary to provide corresponding recommendation services for new users or users with less interactive information. The general solution to the cold start problem is to collect user preference information when a new user first enters the system, and obtain user preferences by explicit feedback. However, in order not to cause users' disgust, the sample of preference information that can be collected is also very limited. Therefore, how to use a small amount of display feedback information to model users is the key issue of this emerging platform recommendation. In a graph composed of user nodes and item nodes, the essence of predicting recommended projects for users is to use existing edges in the graph to predict potential user to project edges. However, the number of existing edges is relatively small (due to the sparse data in rating matrix), resulting in limited prediction performance.

**Figure 1:**
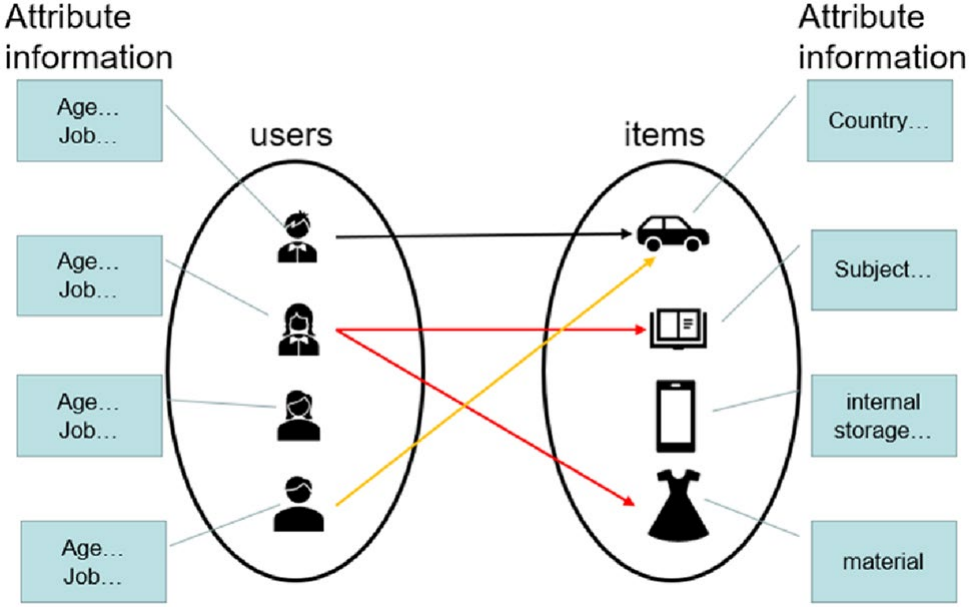
General heterogeneous attribute information network.

Due to its effectiveness and flexibility, network embedding can address the issue of data sparsity and has achieved significant results in various downstream tasks. By treating projects as sets of different attributes, the edge from users to projects can be transformed into multiple edges from users to attributes. Based on the number of node and edge types in the network, recommendation algorithms based on network embedding can be divided into two types: recommendation algorithms based on homogeneous information network embedding and those based on heterogeneous information network embedding. In real-world networks, nodes and edges are not only classified into multiple types, but also have their own attribute information. Networks with multiple types of nodes and attribute information are called heterogeneous attribute information networks. Typically, a heterogeneous attribute information network appears like the one shown in Fig. 1.

The Fig. 1 shown above is a traditional heterogeneous attribute information network consisting of users and items, which includes users, user attributes, items, and item attributes. However, in practical scenarios, there are usually a large number of item attributes while user profiles often have limited content and may involve privacy concerns, making it extremely difficult to model new users.

This article proposes a method for providing recommendations to users with only a small amount of display feedback data, which is aimed at emerging platforms. We define these users as "small-sample users," who may be new users or old users with sparse rating matrices. We propose a recommendation method based on self-attention attribute network embedding, which can directly model small-sample users based on limited data. First, we regard items as sets of different attributes and use self-attention mechanisms to combine attribute representations into compact embeddings in space. We represent users as different anchor vectors in space to learn their different preferences, with each anchor point representing a single preference of the user. When making recommendations for small-sample users, we can efficiently reconstruct them by reusing existing anchor vectors of other users, achieving the goal of fast and accurate recommendations. Overall, our contributions are mainly as follows:

- An attributed network embedding based on self-attention mechanism for recommendation method is proposed.
- More objective attributes from expert comments are extracted for supplementary item documents.
- User information is represented as reusable anchor vectors in space for dealing with cold start.
- Extensive experiments have been conducted on four real-world datasets, and experience has shown that our proposed method performs better.

This paper is organized as follows. In section "Related work", we summarize the related work about our research. In section "Methodology", we define our research problem and present the details of our proposed model. Experimental results are reported in section "Experiments" and the conclusion and discussion are drawn in Section "Conclusion".

## Related work

We briefly summarize this section to self-attention mechanism, network embedding and attributed network embedding.

### Self-attention mechanism

The most representative method based on deep neural networks is the application of CNN and RNN. RNN cannot perform parallel calculations because each cell needs to wait until the information of other cells is input and computed before it can be calculated. CNN can perform parallel calculations, but it needs to stack many layers to capture long-term information. The advantage of the self-attention mechanism is that each unit can capture the information of the entire sentence, and the output can also be calculated in parallel. In the field of recommendation, many scholars have adopted a hybrid neural network model to integrate the advantages of various neural network models and introduced the attention mechanism to give different degrees of attention to the extracted features, thus further improving the recommendation performance. Reference^17^ uses the attention mechanism to combine the local implicit state vector of Bi-LSTM with the topic vector of the global hierarchical Dirichlet process and proposes a classification method of Bi-LSTM Web services based on topic attention.

### Recommendation technology of network embedding

The research of network embedding that has arisen in recent years originates from the representation learning in the field of natural language processing^18^, and has derived network embedding based on factor decomposition^19^, network embedding based on random walk and network embedding based on deep learning. In the early stage, there were many researches on network embedding recommendation technology based on factor decomposition, such as the HOPE algorithm was proposed in Reference^17^ is to decompose multiple asymmetric relational matrices in a directed graph. Influenced by the idea of word2vec in the NLP field, the deep walk proposed by Preozzi et al.^20^ based on the random walk recommendation technology for network embedding is a graph structure data mining algorithm, which can learn the hidden information of the network. With the development upsurge of the deep neural network, scholars combine the deep neural network with the graph embedding, such as Grover et al.^21^ proposed SNDE which uses the automatic encoder to find an embedding node that can reconstruct its neighborhood, and then map the fixed point and space based on the Laplace feature.

Due to network embedding has good scalability and can adapt to large-scale data sets, many scholars combine it with recommendation technology, such as Item2Vec^22^, Prod2Vec^23^, etc. Its principle is to learn the nodes on the item graph into the low-dimensional vector space, thus replacing the traditional item-based collaborative filtering algorithm based on the similarity calculation formula.

### Recommendation technology based on attribute network embedding

**Figure 2:**
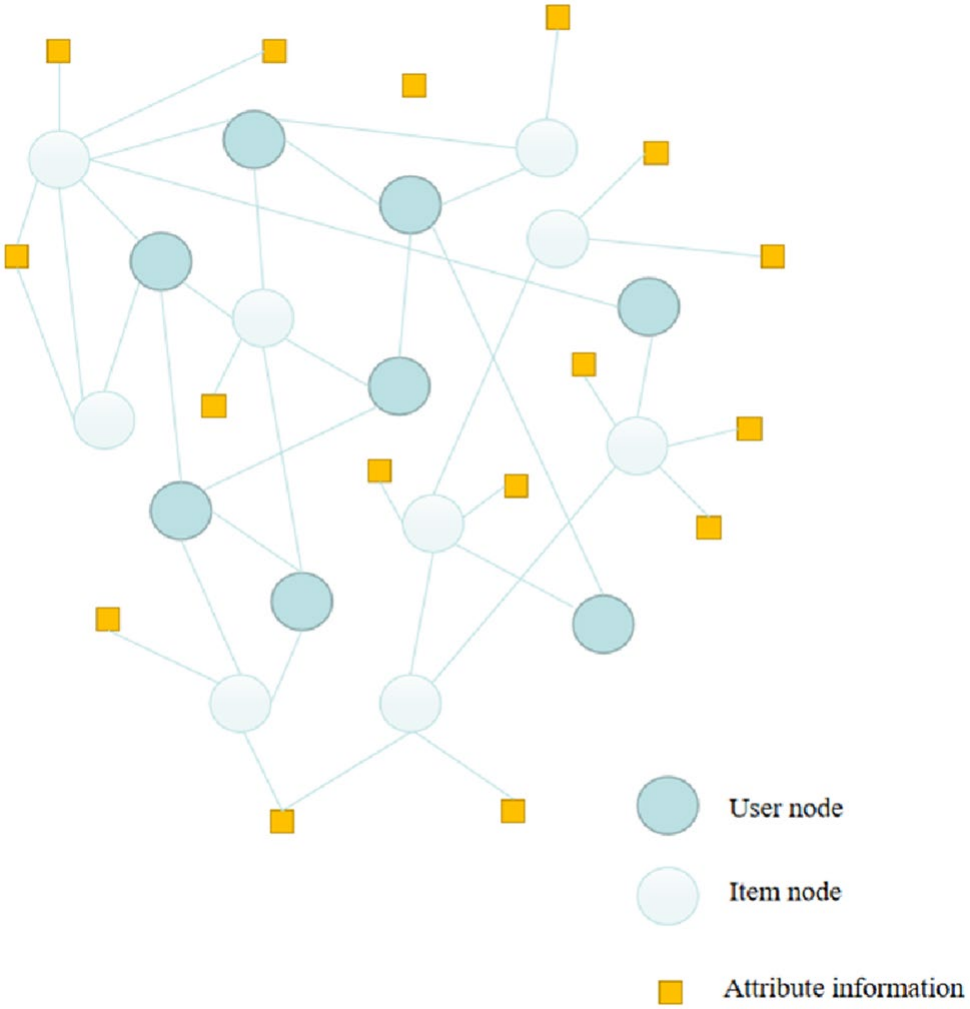
Attribute network diagram.

As shown in Fig. 2 is an attribute information network that contains the attribute information of nodes. The embedding method for processing attribute information network is attribute information network. Attribute network embedding maps the attribute information of nodes to a joint low-dimensional vector space to learn the embedded representation of nodes. MMDW^24^ is a recommended method based on deep walk matrix decomposition and support vector machine^25^, Its learning model combines the label information of nodes. Using the semi-supervised network embedding method of the label information of nodes, Reference^25^ regards the text content as a special node, Construct an enhanced network learning node representation. Reference^26^ proposes a general model of social networks that can integrate structural information and membership information. Reference^27^ uses skip-gram to aggregate the characteristics of neighbor nodes to learn node representation while using neighbor enhanced automatic coder to model node attribute information.

The above methods have effectively improved the performance of the recommendation algorithm, but there is no good improvement effect for users with less explicit feedback information. The recommendation system will have a large number of new users every day and provide a small amount of feedback data in a short time. It is inefficient to retrain new users every time. In order to solve the above problems, this paper proposes an attributed network embedding with self-attention mechanism for recommendation method (AESR). This method is based on multi-head self-attention mechanism and uses attribute information network embedding learning, and then uses matching function to solve the product recommendation task.

### Attribute extraction

Generally, the attributes of items mainly represent the tags of items, but the number of tags is relatively small. In addition, tags are generally provided by the information producer, so the tags are not objective enough. This paper also regards the document information of experts’ comments and item descriptions as supplementary attributes. However, the amount of document information is mostly complicated, so we need to extract topic from document information. Attribute extraction generally adopts LDA model^28^. Firstly, LDA is a well-established topic modeling technique that has been widely used in various natural language processing and information retrieval tasks. It is particularly effective in uncovering latent topics within a collection of textual data, which aligns with our objective of identifying hidden patterns and topics in the dataset. Secondly, LDA offers a probabilistic framework for modeling document-topic and topic-word relationships. This probabilistic nature allows us to capture the inherent uncertainty and variability in textual data, making it suitable for handling diverse datasets. The ability to model topics and their distributions within documents makes LDA a versatile choice for uncovering underlying structures in textual data, regardless of the specific domain or dataset. However, it is important to acknowledge that while LDA can be a powerful tool for topic modeling, its effectiveness may vary depending on the nature of the dataset. Some datasets may exhibit characteristics that make LDA less suitable, such as extremely short documents or an absence of clear topical structures. In such cases, alternative methods or modifications to the LDA model may be considered.

The LDA model is an unsupervised model, which does not require labor costs. It can classify texts with the same semantics according to the frequency of word co-occurrence. The process is as follows:

The comments are preprocessed by removing punctuation, stop words, digits, and converting to lowercase, in order to reduce noise and redundancy in the document. Then randomly assign a topic to all words in all documents, that is as follows:

1 $$z_{m,n} = k\sim Mult\left( {1/K} \right)$$

where $m$ represents the $m$-th document, $n$ represents to the $n$-th word in the document, $k$ represents the topic, $K$ represents the total number of topics. and then the corresponding $n_{m}^{(k)} + 1$, $n_{m} + 1$,$n_{m}^{(t)} + 1$,$n_{k} + 1$, they respectively represent the number of occurrences of $k$ topics in the $m$ document, the sum of the number of topics in the $m$ document, the number of $t$(word) corresponding to $k$ topics, and the total number of words corresponding to $k$ topics.

- Repeat the iteration for the following operations.
- Traverse all words in all the documents ,if $$t$$ is respond to $$k$$ in current document $$m$$,then $$n_{m}^{(k)} - 1$$,$$n_{m} - 1$$$$n_{m}^{(t)} - 1$$$$n_{k} - 1$$,that is, take out the current word first, and then sample a new topic recording to the probability distribution of the topic in LDA, the corresponding $$n_{m}^{(k)}$$,$$n_{m}$$,$$n_{m}^{(t)}$$,$$n_{k} + 1$$ respectively.
- Output the subject word parameter matrix and Documentation—Topic Matrix after iteration
- After removing redundancy from the first f topics and tags obtained, this paper uses them as item attributes. Then, using the bag-of-words model, each attribute is converted into a vector, which becomes the d-dimensional feature of the responding tag.

## Methodology

Our model first extracts item attributes and encodes the attribute set of the item into the item preference space. The preference of different users are different points in the item preference space, and users become the anchor vector in the space. In the item preference space, each anchor vector of the user is used to measure the user's preference for attributes, and the similarity relationship between the anchor vector and the item vector is constructed through metric learning. In order to make the set of anchor vectors of different users can be reused, the user's preferences are decomposed into different anchor vector representations, and each anchor vector is thus decoupled to only focus on the user's single preference. When the feedback data of the target user is collected, the user can be quickly modeled.

### Item self-attention model

Self-attention mechanism is a basic component that can be calculated in parallel which is shown as Formula(2):

2 $$Attenion\left( {Q,V,K} \right) = softmax\left( {\frac{{{\text{QK}}^{T} }}{\sqrt d }} \right)$$

where $Q$ represents query vector, $V$ represents value and $K$ represents key.$softmax( \cdot )$ function is used to normalize the matrix. The self-attention mechanism takes the embedding of items as input, converts them into three matrices through linear transformation, and uses multiple attention, which is shown as Formula (3) and (4):

3 $$O = MHA(E) = concat({\text{head}}_{1} ,{\text{head}}_{2} ...{\text{head}}_{h} )W^{H}$$

4 $${\text{head}}_{i} = Attention(EW^{{^{Q,} }} ,EW^{{^{QV,} }} .EW^{{^{Q,K} }} )$$

where $W^{{^{Q,} }} ,W^{{^{V,} }} ,W^{{^{K,} }}$ represent projection matrices; $E$ represents embedding matrix of all items; $h$ indicates the number of heads. The choice for the dimensions in this paper is $d_{k} = d_{v} = d/h$.This paper use Point-Wise Feed-Forward Networks(FFN) to strengthen the nonlinear model which defined as Formula (5):

5 $$F = FFN(SelfAttn( \cdot ))$$

Due to an item generally has many attributes, it can be seen as a combination of different attributes, and the content that users are interested in can be understood as a combination of different item attributes. For example, some users like "horror thrillers" and another like "horror comedies". Given an item $I$ can be represented as a set of attributes $I = \{ A_{1} , \cdots ,A_{n}^{T} \} \in R^{n \times d}$, $A_{{_{k} }}$ represents the embedding of the d-dimension feature of the attribute in item $I$, and ${A}^{T}\in {R}^{n\times d}$ represents the attribute feature. The item attribute network defines a set mapping relationship $g:R^{n \times d} \to R^{d}$, which maps the $n$ attributes of the item to a low-dimensional embedded $p \in R^{d}$ in the item preference space. Given $n$ attribute characteristics $A$ of the item, we calculate their output under multi-headed self-attention as Formula (6) and (7):

6 $$SelfAttn(A)\; = LayerNorm\left( {H + \sigma \left( H \right)} \right)$$

7 $${\text{where}}\;H = LayerNorm(A + MutiHead(A,A,A))$$

For an item $I$, the attribute size is not fixed, and the attribute order does not affect the representation of the item. Therefore, $g( \cdot )$ needs to be able to handle items with different attribute quantities, and has sorting invariance. For the feature representation $A$ of a given attribute, the multi-level self-attention function is used as the encoder of the item. The form is shown as Formula (8):

8 $$T = FFN(SelfAttn_{ \times M} (A)) \in R^{n \times d}$$

AESR improves the performance of feature learning by stacking multi-layer self-attention functions. The order of output $T$ and input $A$ of multi-layer self-attention is one-to-one. The method based on self-attention can extract the correlation between the current sample and other samples, so that the feature representation of each sample contains context information.

In order to obtain the compact depth representation of items, the obtained self-attention output *F* is mapped *n* samples to a single sample by using muti-head attention mechanism:

9 $$v = LayerNorm\left( {h + \sigma \left( h \right)} \right)$$

10 $${\text{where}}\;h = LayerNorm(\rho + MultiHeads(s,T,T))$$

where $\rho \in R^{d}$ is learnt as parameter of the model, $v$ is the presentation of item.

### Matching function

**Figure 3:**
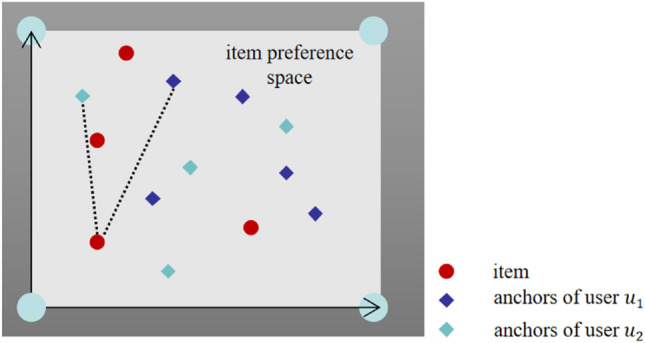
Schematic diagram of item preference embedding space.

AESR regards users as a set of learnable parameters. Since different users may have different preferences, each user is represented as a set of anchor vectors distributed in the item preference space. Figure 3 shows a simple diagram.

In Fig. 3, user ${u}_{1}$ and ${u}_{2}$ are described by two sets of anchor vectors. Each anchor represents a single preference of responding user. The similarity of two users’ preference can be expressed by the similarity between two groups of anchors. When users with less explicit feedback data enter the system, they can quickly reuse features and model them.

$(U_{i} )_{m}$ represents the anchor vector set of *m* different users, where $U_{i} = [u_{i1} ,...,u_{i\rho } ]$, which represents $\rho$ anchors of $i$-th user. $u_{ik} \in R^{d}$ is the $k$-th anchor. The preferences of user can be described by $\rho$ learnable anchors in space, so we call these learnable anchors as l-anchors, and call the corresponding model AESR-L.

To obtain the vector $U_{i}$ that represents a user's preferences and interests, various techniques and algorithms can be used to generate it. The most common method is based on collaborative filtering, which recommends other users or items similar to the target user based on their historical behavior and rating data. In terms of project attributes, this article considers expert comment information, so from the perspective of the target user's preferences, this article also considers the target user's comment information. The steps are as follows:

1. Collect the target user's comment and rating data. Same as section "Attribute extraction", first preprocess the comment information, including removing punctuation, stop words, numbers, and lowercase conversion, to reduce noise and redundant information in the document.
2. Convert the comment and rating data into vector representations as follows:

- Assuming there is a comment document $C$ consisting of $n$ words, represented as $C = \{ w_{1} ,w_{2} ,...w_{n} \}$, where $w_{i}$ represents the *i*-th word in the document. To use the bag-of-words model, where the document is represented as a *V*-dimensional vector, where *V* is the size of the vocabulary and each dimension of the vector represents the count of a word in the document.
- Let $x_{i}$ denote the count of the *i*-th word in the vocabulary in $C$. Then, the vector representation of $C$ is: $x_{C} = [x_{1} ,x_{1} ,...x_{|V|} ]$
- For user rating data, let's assume there are $m$ ratings, denoted as $r = \{ r_{1} ,r_{2} ,...r_{m} \}$, where $r_{i}$ represents the rating given by the user for a certain item. To convert the rating data into a vector representation, the rating values can simply be regarded as the elements of the vector: $x_{r} = \{ r_{1} ,r_{2} ,...r_{m} \}$

3. For each user, take the average of all their comment and rating vector representations to obtain the target user's average vector.
4. Use DBSCAN (Density-Based Spatial Clustering of Applications with Noise) to cluster all users' average vectors and obtain some clustering centers. DBSCAN belongs to the density clustering method, which defines clusters as areas with sufficient high density and is suitable for clusters of various shapes and sizes. This is very useful for discovering irregular user groups in the data. These clustering centers can be viewed as anchor points representing different user preferences.
5. For each user, calculate the distance between their average vector and all anchor points. The anchor point with the smallest distance is the user's anchor vector, representing their partial preferences.

As shown in Formula (10), a given embedding of item ${v}_{j}$, the similarity between ${v}_{j}$ and all l-anchors ${U}_{i}$ is calculated as Formula(11):

11 $$\begin{gathered} r_{ij}^{L} = F(u_{i} ,v_{j} ) = \frac{1}{\rho }\sum\nolimits_{k = 1}^{\rho } {u_{ik}^{T} } v_{j} \hfill \\ s.t.||u_{i} k|| = ||v_{j} ||_{2} = 1 \hfill \\ \end{gathered}$$

It can be seen from above that the AESR-L model requires each user to interact with enough items to reliably learn l-anchors. However, when there are not enough items in the system, there is less data available for training, which can not be fully learned and represented. To solve this problem, we define a set of learnable vectors to learn the average preference of items among all users. For user, expand the corresponding user parameter matrix to the following form:

12 $$U_{i}^{P} = [U_{i} ,P]$$

where $U_{i}$ contains all personalized anchor vectors of the user, and all users share the vectors in $P$. $\rho_{1}$ and $\rho_{2}$ are the number of different anchor vectors. The vector in $P$ is called p-anchors, and the corresponding model is called AESR-p. The rating prediction based on the p-anchors is calculated as Formula (13):

13 $$\begin{gathered} r_{ij}^{P} = \frac{1}{{\rho_{1} }}\sum\nolimits_{k = 1}^{{\rho_{1} }} {u_{ik}^{T} v_{j} + } \frac{1}{{\rho_{2} }}\sum\nolimits_{k = 1}^{{\rho_{2} }} {u_{ik}^{T} v_{j} } \hfill \\ s.t.||u_{i} k||_{2} = ||\rho_{k} || = ||v_{j} ||_{2} = 1 \hfill \\ \end{gathered}$$

where, $\rho_{k}$ represents the ${\text{k}}$ average anchor in P. If there are enough items, user preferences can be well captured without an average anchor, so LPAE—*u* and LPAE—*g* will have similar performance in this case. However, when the training data is insufficient, the average anchor can improve the recommendation effect.

AESR uses nonoverlap promote variable selection ^29^ to regularize the anchor vector of each user. This method can promote the non-overlapping effect in variable selection. In the attribute modeling task, we hope that there is less overlap between the attribute features selected for different responses. The vector is more "targeted", assuming that L is l-anchor, and the regularization calculation is shown as Formula(14):

14 $$\Omega (W) = tr(L) - log\;det(L) + \gamma \sum\limits_{i = 1}^{m} {|w_{1} |_{1} }$$

where $\gamma$ is the trade parameters between the two regularizes and $L$ is one of the anchor matrices.

**Figure 4:**
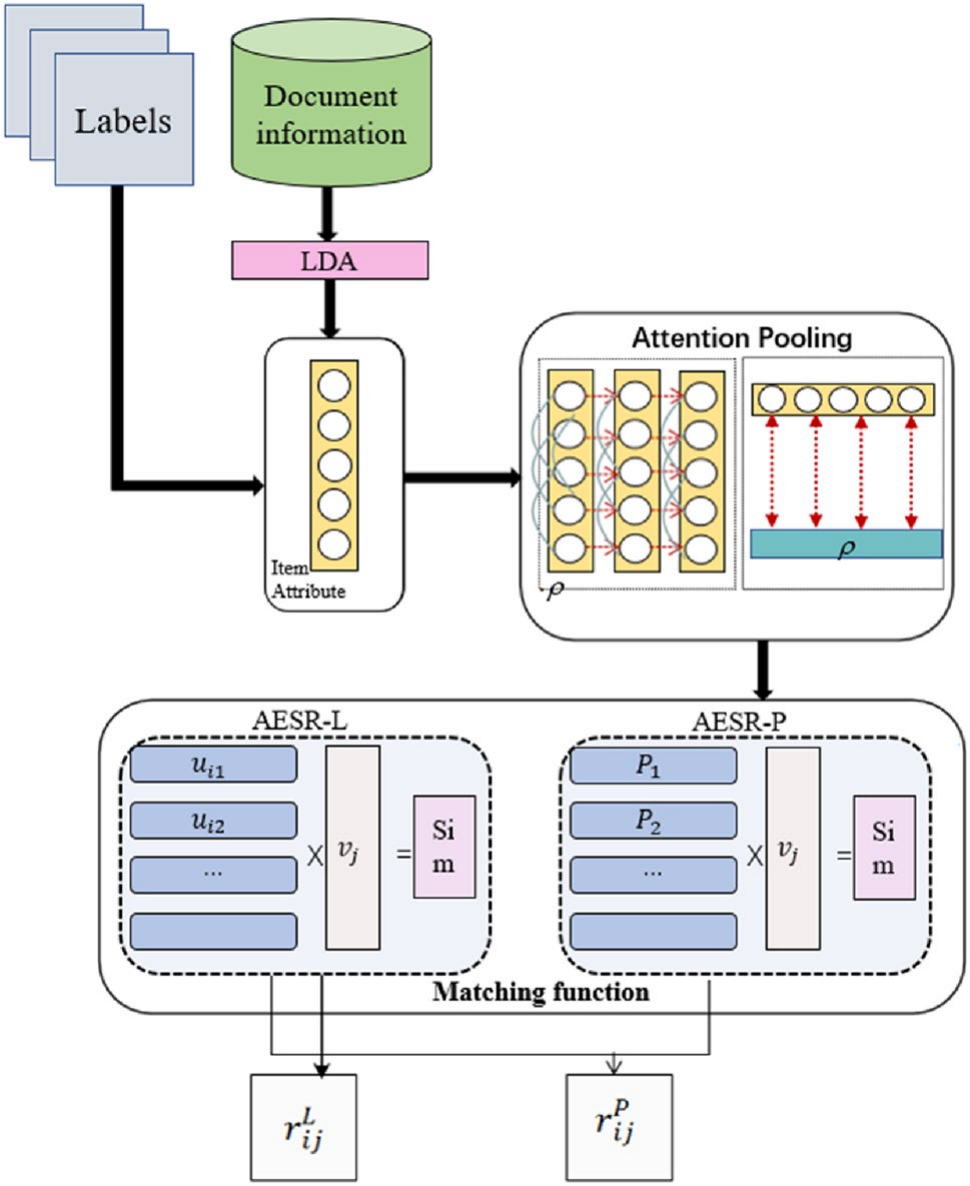
The overall framework of AESR.

Figure 4 shows the overall framework of AESR.

### Recommendation for small sample users

The above method we adopt can meet the needs of recommendation for users. By metric learning, users can pull in their favorite items and learning anchor vectors, so the anchor vectors between users with similar preferences will also be pulled closer. Therefore, when the user only displays a small amount of feedback data, the user can be analyzed by reusing the anchor vector of other users.

For $V$ which is the item set of the given target user ${u}_{T}$, the number of elements in $V$ is very small. Wherein, $v_{j}$ represents the feature representation of the *j* item in the item preference space. Combining with optimization based strategy, This paper adopts an anchor vector reuse strategy for a small sample user modeling. That is, analyze a small sample of users by reusing their partial preferences.

Let $ANC = \{ (i,k)\}$ represents the index set of user's anchor vectors, where the size of the set is equal to the number of user's anchor vectors, that is,$|ANC|=\rho$

15 $$ANC_{{\;u_{T} }} = \mathop {\arg \max }\limits_{{(u_{ik} )_{i,k} }} \sum\limits_{{v_{j} \in V}} {\sum\limits_{(i,k) \in ANC} {u_{i}^{T} v} }$$

Based on the strategy of anchor vector reuse, the first $\uprho$ anchor vectors most relevant to the target user feedback $V$ are calculated from all user anchor vectors to represent the ${u}_{T}$. In particular, when $|V| = 1$, all anchor vectors which cosine similarity with items greater than 0 are used to reduce uncertainty.

### Optimization function

This paper uses the binary cross entropy (BCE) index as a loss function to evaluate the loss of each algorithm. The formula is as Formula (16):

16 $$Loss = - \frac{1}{N}\sum\limits_{i = 1}^{N} {y_{i} \log (p(y_{i} ))} + (1 - y_{i} )\log (1 - p(y_{i} ))$$

where $N$ represents the total training sample size, $y_{i}$ represents the real tags, and $1 - y_{i}$ represents the predicted tags.

## Experiments

The local experimental environment is shown in Table 1.

**Table 1:** Local experimental environment.

Environment	Edition
Python	3.7.2
Tensorflow	2.6.2
Keras	2.6.0

### Experiment settings

#### Datasets

The data set in this paper adopts movieLens(20m), FilmTrust and Epinions, which can only use tags as attributes, and Amazon, which can extract attributes from text and comments. The following is an introduction of these data sets:

The statistical table of the datasets is shown as Table 2.

**Table 2:** The details of datasets.

Data set	Users	Items	Ratings
movieLens(20M)	138,493	27,278	20,000,263
FilmTrust	1,641	2,071	35,497
Epinions(665k)	49,290	139,738	664,824
Amazon Book	70,697	24,915	2,557,746

This paper first preprocesses the above dataset, retaining user data with both rating and label information in the original dataset. In the experiment, we randomly divided the entire dataset into 80% for training, 10% for validation, and the remaining 10% for testing.

#### Comparison method

In order to verify the performance of the proposed method, we designed and implemented a comparative experiment with the following methods.

- **MF:** Matrix factorization is the most widely used collaborative filtering model in recommendation systems. It is to decompose a matrix into two or more matrices, so that the decomposed matrix can be multiplied to obtain the original matrix.
- **PMF**(Probabilistic Matrix Factorization)^20^: The core idea of PMF is that the relationship between users and items can be determined by linear combination of several factors. PMF takes the maximum posterior probability of the potential features of users and items as the objective function, obtaining the estimated value of the potential feature vectors. Then PMF predicts the unknown rating through its inner product.
- **SoRec** (Social Recommendation Using Probabilistic Matrix Factorization)^4^: The social relationship matrix is added on the basis of PMF and social information is integrated.
- **Deepwalk:** Deepwalk uses the co-occurrence relationship between nodes in the graph to learn the vector representation of nodes.
- **MMGCN:** It is a graph-based algorithm. To learn user preferences for different modalities, it constructs user-item interaction graphs separately for each modality. Finally, it accumulates the learned user-item representations from different graphs to obtain the final user-item representation, which is then fed into the prediction module.
- **MPGAT:** Similar to MMGCN, it is also a graph-based algorithm. It also constructs user-item interaction bipartite graphs for different modalities. However, unlike MMGCN, it incorporates a GRU gating module during the training process of individual modality graphs. It also utilizes attention mechanisms to aggregate neighbor information, resulting in better vector representations.

#### Parameter settings

We set the latent dimensionality to 128 for all methods. For the ASER-L method, we employed a setting with $\rho$ = 64, while for the ASER-P method, we used $\rho_{1}$ = $\rho_{2}$ = 32, respectively. All algorithms utilized the SGD^30^ as the gradient descent algorithm, with the learning rate decaying when the performance no longer improved.

### Experiment results and analysis

The recommendation task studied in this paper will recommend the top-K non-interactive items with high matching degree with user preferences to users. It focuses on whether the items that users really like are in the recommendation list, so this paper uses AUC to measure the accuracy of the model. The normalized discount cumulative gain (NDCG) is used to reflect the ranking quality of the model. The calculation method for these two evaluation indicators are shown in Formula (17) and (18):

17 $${\text{AUC}} = \frac{{\sum\nolimits_{{i\in{\text{positive}}}} {{\text{rank}}_{i} - \frac{M(1 + M)}{2}} }}{M \times N}$$

18 $$NDCG = \frac{{\sum\limits_{i = 1}^{p} {\frac{{2^{{rel_{i} }} - 1}}{{\log_{2} (i + 1)}}} }}{IDCG}$$

where $rel_{i}$ represents the correlation rating of the recommendation results at position $i$, $IDCG$ represents the list of best recommendation results returned by the recommendation system for a user, and $p$ represents the length of the recommendation list to be examined. If the positions of the correctly recommended items are higher, the NDCG value will be higher.

- The default recommended list length K in this article is 10. We first compare AUC and NDCG as follows.

It can be seen from the tables above that the model in this paper is superior to other comparison models in terms of the two evaluation indicators of four datasets (Tables 3, 4); The effect of Deepwalk is better than that of MF. PMF and SoREC. The reason may be that Deepwalk is a deep learning model, which is suitable for learning complex matching functions in recommendation problems, can approximate continuous functions and achieve better results. MGAT and MMGCN outperform DEEPWALK in terms of recommendation performance for several reasons. Firstly, they employ more complex model architectures, including techniques such as Graph Attention Networks (GAT) and Gated Recurrent Units (GRU). These enhancements enable them to better capture high-order neighbor information and local semantic context within the graph, leading to a more accurate understanding of user behaviors and interests. Secondly, both MGAT and MMGCN effectively integrate multi-modal information, allowing them to consider different data types simultaneously, such as text and images, resulting in a more comprehensive understanding of user interests. Additionally, they increase model flexibility by decomposing user preferences into different anchor vector representations, making them better suited to adapt to varying user requirements and deliver more personalized recommendations. However, regarding why MGAT outperforms MMGCN, it relates to differences in model structures, a conclusion that aligns with the findings in Reference^16^. In summary, these factors collectively contribute to MGAT and MMGCN surpassing DEEPWALK in terms of recommendation performance and, in some aspects, making MGAT perform even better.

**Table 3:** Comparison of AUC/NDCG of each algorithm on top-K recommendation tasks.

Methods	Film trust		Epinions		movieLens		Amazon Book	
	AUC	NDCG	AUC	NDCG	AUC	NDCG	AUC	NDCG
MF	0.7602	0.3365	0.7933	0.3510	0. 7752	0.3354	0.7238	0.3321
PMF	0.7771	0.3422	0.7891	0.3502	0.7823	0.3507	0.7543	0.3324
SoRec	0.7594	03.304	0.7877	0.3392	0.8527	0.4954	0.7523	0.3312
Deepwalk	0.8972	0.5078	0.8925	0.5032	0.8796	0.5012	0.7954	0.3490
MMGCN	0.9095	0.5123	0.9080	0.5114	0.9002	0.5767	0.8237	0.4694
MPGAT	0.9129	0.5195	0.9094	0.5140	**0.9130**	**0.6043**	0.8596	0.4799
AESR-L	**0.9201**	**0.5358**	**0.9187**	**0.5245**	0.9023	0.5945	**0.8892**	**0.5023**
AESR-P	**0.9201**	**0.5358**	0.9183	0.5237	0.9025	0.5941	0.8634	0.5003
%Improv	0.88%	0.39%	0.01%	2.04%	− 1.15%	− 0.17%	3.44%	4.67%

The best values are highlighted in bold.

**Table 4:** Comparison of AUC/NDCG of each algorithm on top-K recommendation tasks for small sample users.

Methods	Film trust		Epinions		movieLens			Amazon Book	
	AUC	NDCG	AUC	NDCG	AUC	NDCG		AUC	NDCG
MF	0.7582	0.3245	0.7527	0.3438	0.7238	0.3378		0.7018	0.3021
PMF	0.7764	0.3422	0.7956	0.3543	0.7543	0.3243		0.7506	0.3224
SoRec	0.7585	0.3304	0.7833	0.3423	0.7523	0.3231		0.7512	0.3307
Deepwalk	0.8421	0.4773	0.8652	0.4141	0.7954	0.3489		0.7688	0.3457
MMGCN	0.8434	0.4807	0.8691	0.4178	0.8534	0.4897		0.8323	0.4428
MPGAT	0.8455	0.4821	0.8697	0.4181	0.8891	0.4306		0.8567	0.4869
AESR-L	0.8426	0.4844	0.8722	0.4292	0.8892	0.4312		0.8501	0.4823
AESR-P	**0.8577**	**0.4877**	**0.8891**	**0.4334**	**0.8934**	**0.4425**		**0.8614**	**0.4883**
%Improv	1.44%	1.16%	2.23%	3.67%	0.48%	2.76%		0.54%	0.29%

The best values are highlighted in bold.

ASER outperforms all others on most datasets, with the exception of the Movielens dataset where it falls slightly behind MPGAT. This discrepancy can be attributed to the high density of the Movielens dataset. In dense datasets, ASER may not demonstrate its full potential when recommending to regular users. However, when it comes to recommending to small-sample users, AESR consistently delivers the best performance across all four datasets. Notably, the Epinions dataset shows the most significant improvement, likely due to its sparse nature. These findings are in line with our expectations.

- Since we adopt the multi-head self-attention stacking mechanism, we compare the number of self-attention heads in AESR-L which is shown in Fig. 4.

**Figure 5:**
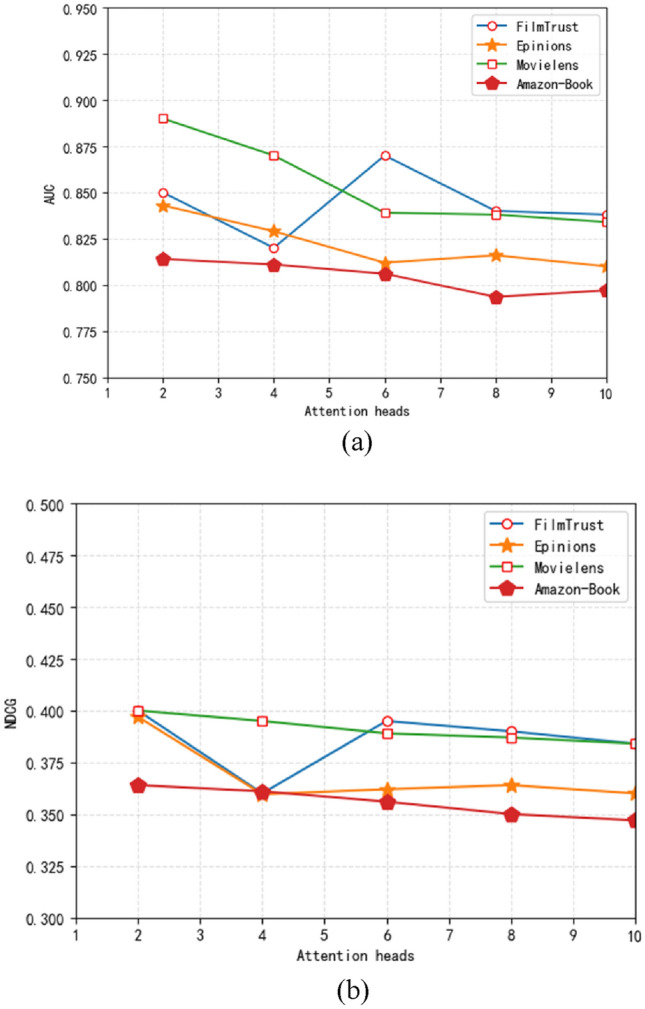
The influence of attention heads number on NDCG.

It can be seen from the figure that too large attention head makes a single attention dimension too small, thus limiting the expression ability of attention. The optimal attention header of the Filmtrust is 6 and the Epinions is 8. When the head of attention is greater than or less than the optimal value, the performance decreases slightly (Fig. 5).

**Figure 6:**
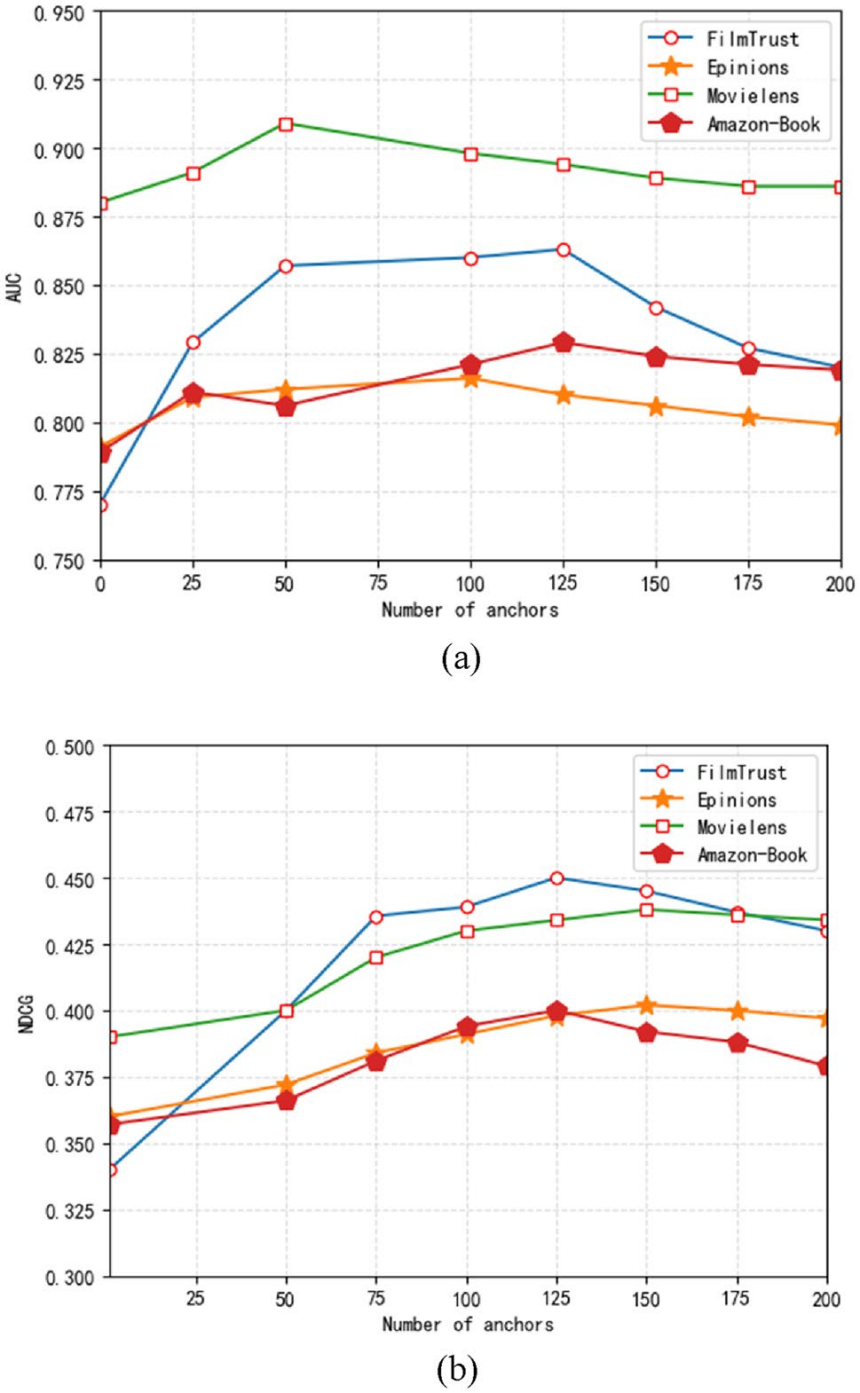
The influence of anchor number on NDCG.

- In order to show the effect of different number of anchors, we train AESR-L with different number of anchors. The results are shown in Fig. 6.

When the number of anchors is 1, AESR attempts to embed and cluster all items into a class for each user, thus losing a lot of information, so the performance is as poor as expected. As the number of anchors increases, the performance improves significantly. However, when the number of anchors becomes larger than the required optimal number of clusters, the performance begins to decline.

**Figure 7:**
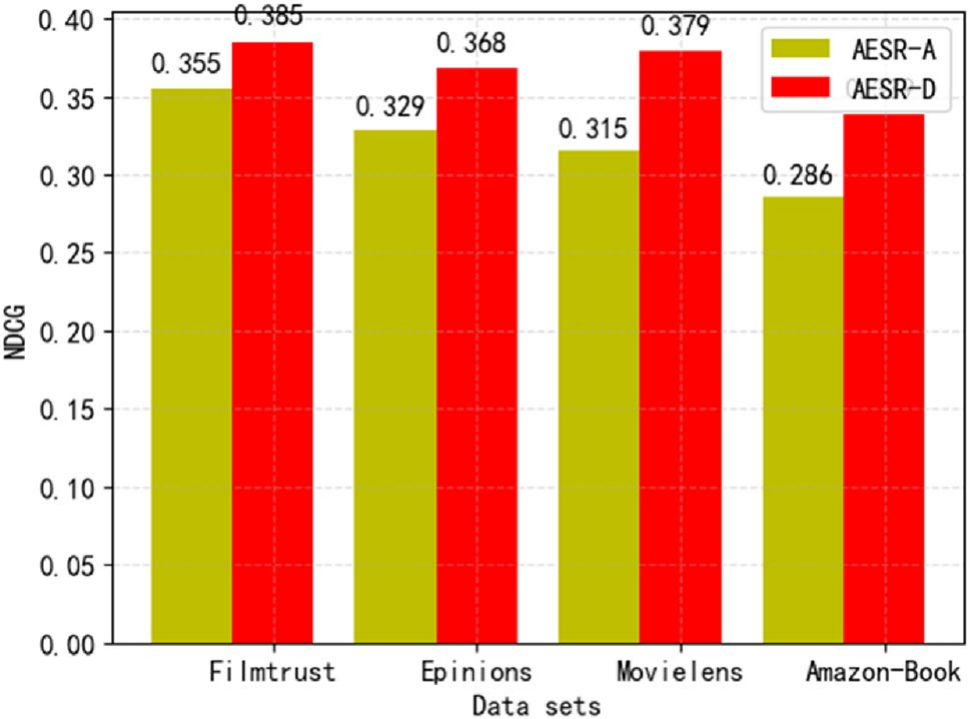
The importance of documents of experts’ comments and item introduction.

- In order to verify that adding expert comments and project documents when selecting attributes is meaningful, we conducted the following ablation experiments. AESR-A represents the model that only uses labels as attributes, while AESR-D represents the model that uses both labels and documents as attributes. The number of anchors selected was 125. The experimental results are as Fig. 7.

We can find that adding project attributes through documents makes sense, especially for Amazon Book, as there is a large amount of document information in this dataset.

- Comparison with baselines on top-K recommendation

**Figure 8:**
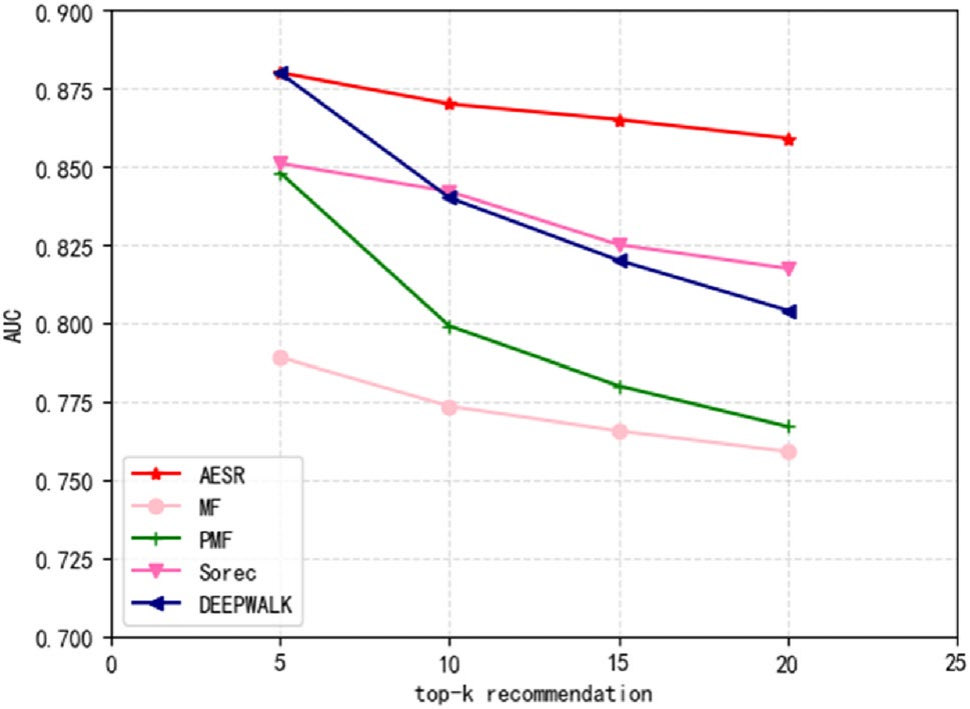
Comparison between AESR and baselines in terms of AUC.

Our final task is to recommend potential projects to users. Therefore, in this experiment, we compared AESR baseline models on top-k recommendation which use movieLens dataset. The experimental results are as Fig. 8.

The larger the number of recommendations, the more items the model recommends, but it also increases the noise and inaccuracy of recommendations. The relationship between recommendation quantity and AUC depends on the specific implementation of the recommendation algorithm. Generally speaking, an increase in the number of recommendations may lead to a decrease in AUC, as an increase in the number of recommendations makes the discriminant ability of the classifier more difficult. However, if the recommendation algorithm can maintain high-quality recommendations while recommending more items, the AUC value may also increase. From the experimental results, we can see that AESR decreases more slowly with the increase of recommendation quantity, indicating that our model has better judgment ability.

### Discussion

Based on the experimental results, it is evident that AESR outperforms other network structure-based embedding models in the final recommendation task. This observation strongly underscores the effectiveness of our proposed approach. However, our approach is better suited for emerging platforms with a significant number of "small-sample users" and limited information. In scenarios with limited data, AESR excels at quickly modeling users and responding to their needs. Nevertheless, it has its limitations, as it cannot capture fine-grained user preferences and may not perform as well on mature platforms. In future research, we plan to focus on uncovering fine-grained user preferences to better adapt to user demands.

## Conclusion

To address the problem that recommendation performance is greatly affected when users do not have enough explicit feedback data, in this work we proposed a recommend model named AESR, which is designed based on attribute network embedding. Firstly, the model uses self-attention mechanism to represent the combination of item attributes as compact embeddings and represents users as multiple anchor points in the item space. Then, the model establishes the correlation between users and items through metric learning. Extensive experiments and data analysis on the real-world data set authenticate that it has been proven that AESR can quickly model in the face of cold start or limited explicit feedback from users without affecting the user experience, significantly improving recommendation performance. In addition, the attention mechanism effectively improves the recommendation performance, further confirming the effectiveness of the proposed model in this article.

## Data Availability

The data that support the findings of this study are available on request from the corresponding author, upon reasonable request.
